# Ochronotic arthropathy mimicking degenerative osteoarthritis in an older adult with alkaptonuria: diagnostic and perioperative lessons from a case report

**DOI:** 10.3389/fmed.2026.1835852

**Published:** 2026-06-29

**Authors:** Yong-Ming Zhang, Ji-Xin Chen, Lei Wan, Feng Ye

**Affiliations:** Department of Orthopedics, Shaoxing Shangyu District Hospital of Traditional Chinese Medicine, Shangyu, Zhejiang Province, China

**Keywords:** alkaptonuria, case report, degenerative osteoarthritis, ochronosis, ochronotic arthropathy, total hip arthroplasty, total knee arthroplasty

## Abstract

**Background:**

Alkaptonuria is a rare inherited metabolic disorder caused by impaired homogentisic acid metabolism. Ochronotic arthropathy is a late musculoskeletal manifestation and may mimic ordinary degenerative osteoarthritis in older adults. This diagnostic overlap may delay recognition of the underlying systemic metabolic disease.

**Case presentation:**

A 67-year-old woman presented with progressive pain and restricted motion of the left knee and hip for four years. Preoperative imaging suggested advanced degenerative arthropathy. However, she had a long-standing history of urine darkening after standing and imaging evidence of multi-joint and spinal involvement. During left total knee arthroplasty, diffuse black-brown pigmentation of the articular cartilage and synovium was observed. Histopathological examination demonstrated ochronotic pigment deposition. The patient subsequently underwent staged left total knee and total hip arthroplasty. At five-year follow-up, knee and hip function were satisfactory, and radiographs showed stable prosthesis positioning without loosening.

**Conclusion:**

Ochronotic arthropathy should be considered in older patients with atypical multi-joint degenerative changes, particularly when urine darkening, spinal involvement, or intraoperative black-brown cartilage pigmentation is present. Although biochemical or genetic confirmation is ideal, clinicopathological recognition remains important. Staged joint arthroplasty may provide durable pain relief and functional improvement in advanced ochronotic arthropathy.

## Introduction

Alkaptonuria is a rare autosomal recessive metabolic disorder caused by pathogenic variants in the HGD gene, which encodes homogentisate 1,2-dioxygenase. Deficiency of this enzyme impairs the catabolism of phenylalanine and tyrosine, resulting in excessive accumulation and urinary excretion of homogentisic acid (1). After exposure to air, homogentisic acid undergoes oxidation, leading to the characteristic dark discoloration of urine (2). Over time, oxidized homogentisic acid-derived pigment deposits in collagen-rich connective tissues, including cartilage, tendons, ligaments, and intervertebral discs, resulting in ochronosis and progressive ochronotic arthropathy (3).

Ochronotic arthropathy usually develops later in life and commonly affects the spine, hips, knees, and shoulders. Because degenerative osteoarthritis is common in older adults, ochronotic arthropathy may be overlooked when clinicians focus only on joint pain, stiffness, joint-space narrowing, subchondral sclerosis, and osteophyte formation (4). Accurate recognition is important not only for explaining atypical multi-joint degeneration but also for systemic evaluation, patient education, long-term follow-up, and family counseling.

Here, we report a case of alkaptonuria-associated ochronotic arthropathy in a 67-year-old woman who presented with apparent degenerative disease of the left knee and left hip. The case highlights diagnostic clues of ochronotic arthropathy in an older adult, perioperative considerations during arthroplasty, and five-year functional outcomes after staged total knee and total hip arthroplasty.

## Case presentation

### Chief complaints

A 67-year-old woman presented with progressive pain and restricted motion of the left knee and left hip for four years, with marked worsening during the preceding six months.

### History of present illness

**Figure 1:**
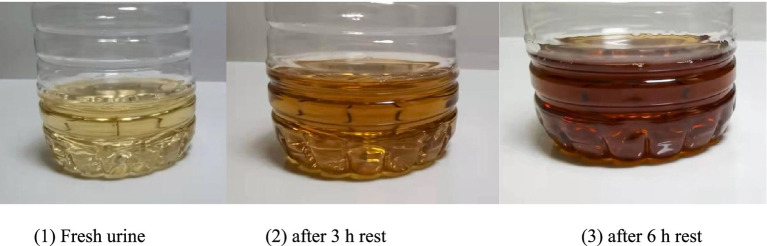
Picture of urine color in patients with dark urine.

Four years before admission, the patient developed insidious pain in the left hip and knee without trauma or an identifiable cause. The pain was aggravated by prolonged walking and relieved by rest. She did not seek medical treatment initially because her daily activities were only mildly affected. During the six months before admission, the pain progressively worsened and was accompanied by restricted joint motion and difficulty walking (Figure 1).

### History of past illness

**Figure 2:**
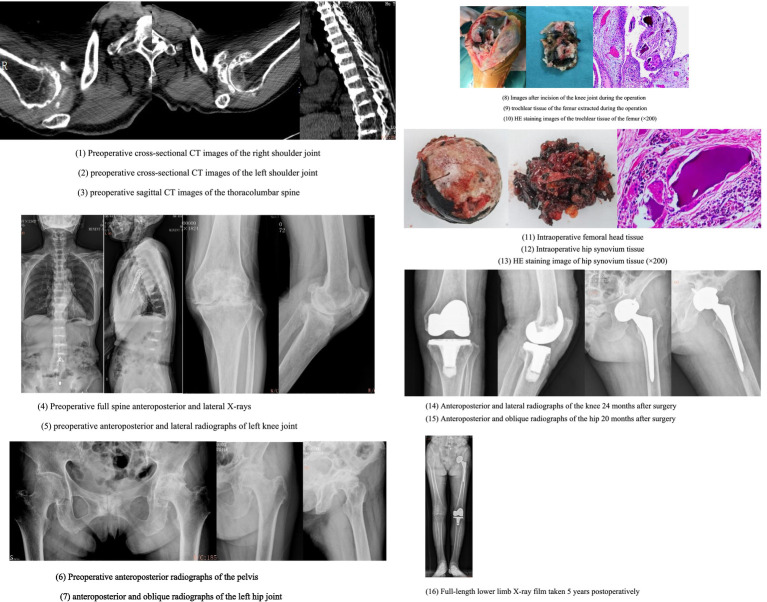
Imaging, intraoperative, pathological, and postoperative findings of alkaptonuria-associated ochronotic arthropathy treated with staged knee and hip arthroplasty.

The patient had a history of intermittent low back pain without regular treatment. Since early adulthood, she had noticed that her urine gradually became dark brown to black after standing for several hours. She also reported dark staining of her underwear. However, repeated routine urinalysis had been normal, and no further metabolic evaluation had been performed (Figure 2).

### Personal and family history

She was of Han Chinese ethnicity. Her parents were non-consanguineous, and there was no known family history of similar symptoms or hereditary disease. Her two sons were healthy.

### Physical examination

Physical examination showed thoracolumbar kyphosis and restricted spinal motion. No obvious pigmentation was observed in the auricles, sclerae, or skin. The left knee showed mild valgus deformity and restricted flexion to 90°, with a preoperative Hospital for Special Surgery knee score of 40. The left hip also showed markedly restricted range of motion, with flexion to 70°, extension to 5°, external rotation to 10°, internal rotation to 5°, adduction to 10°, and abduction to 15°. The preoperative Harris hip score was 43.

### Laboratory examinations

Fresh urine was light yellow but gradually turned brown after standing for 3 h and dark brown after 6 h. Routine blood tests, C-reactive protein, erythrocyte sedimentation rate, human leukocyte antigen-B27, rheumatoid factor, antistreptolysin O, and routine urinalysis showed no abnormal findings. Urinary homogentisic acid quantification and HGD genetic testing were not performed.

### Imaging examinations

Computed tomography images of both shoulders showed degenerative changes, including osteophyte formation and irregular subchondral sclerosis around the humeral heads and glenoids. Sagittal computed tomography images of the thoracolumbar spine and anteroposterior and lateral radiographs of the lumbar spine showed thoracolumbar kyphosis, narrowing of multiple intervertebral spaces, diffuse intervertebral disc calcification, endplate sclerosis, and marginal osteophyte formation, consistent with characteristic spinal involvement of ochronosis. Anteroposterior and lateral radiographs of the left knee showed joint-space narrowing, subchondral sclerosis, and osteophyte formation involving the tibial plateau, femoral condyles, intercondylar eminence, and patella, suggesting advanced degenerative arthropathy. Pelvic radiographs and anteroposterior and oblique radiographs of the left hip showed narrowing of the left hip joint space, subchondral sclerosis, acetabular osteophyte formation, and degenerative changes of the left femoral head.

### Final diagnosis

Based on the long-standing history of urine darkening after standing, multi-joint and spinal imaging findings, intraoperative black-brown pigmentation of cartilage and synovium, and histopathological evidence of ochronotic pigment deposition, the final clinicopathological diagnosis was alkaptonuria-associated ochronotic arthropathy involving the left knee and left hip, with multi-joint and spinal involvement.

### Treatment

The patient underwent left total knee arthroplasty in June 2020. During surgery, diffuse black-brown pigmentation was observed in the femoral condyles, tibial plateau, patellar articular surface, and synovium. Histopathological examination showed chronic synovitis with abundant brown-black ochronotic pigment deposition and focal lymphocytic infiltration. No definitive pathological features of pigmented villonodular synovitis or tenosynovial giant cell tumor were identified. The patient recovered well after total knee arthroplasty and subsequently underwent left total hip arthroplasty in October 2020. Intraoperatively, black-brown pigmentation was observed at the acetabular rim, femoral head cartilage, and joint capsule. The femoral head and synovial tissue were collected for pathological examination. Histopathological examination showed chronic synovitis with extensive ochronotic pigment deposition and foreign-body reaction in the surrounding tissue.

### Outcome and follow-up

The patient was discharged two weeks after surgery and was followed for more than five years, with satisfactory outcomes. After total knee arthroplasty, the Hospital for Special Surgery knee score improved to 62, 78, 86, 89, and 92 at 1 month, 4 months, 10 months, 2 years, and 5 years, respectively. Anteroposterior and lateral radiographs of the left knee obtained 24 months after surgery showed satisfactory prosthesis positioning. After total hip arthroplasty, the Harris hip score improved to 72, 90, 92, and 94 at 3 months, 12 months, 2 years, and 5 years, respectively. Anteroposterior and oblique radiographs of the left hip obtained 20 months after surgery showed satisfactory prosthesis positioning. At the latest follow-up, more than five years after surgery, imaging demonstrated that both the left knee and left hip prostheses remained well positioned, without evidence of loosening. The patient reported minimal pain in the left knee and hip, a normal gait, and independence in daily living. Degenerative changes were also observed in the contralateral knee and hip at the final follow-up, although they had little impact on functional activity.

## Discussion

Alkaptonuria is a rare autosomal recessive metabolic disorder caused by pathogenic variants in the HGD gene, which encodes homogentisate 1,2-dioxygenase. Deficiency of this enzyme interrupts phenylalanine and tyrosine catabolism, resulting in excessive accumulation of homogentisic acid (HGA). HGA is excreted in urine and undergoes oxidation after exposure to air, causing the characteristic dark discoloration of urine. Over time, oxidized HGA-derived pigment deposits in collagen-rich connective tissues, including cartilage, tendons, ligaments, and intervertebral discs, leading to ochronosis and progressive ochronotic arthropathy. The pathogenesis of ochronotic arthropathy is closely related to oxidation and polymerization of HGA-derived metabolites in cartilage and other connective tissues (5). Ochronotic pigment deposition alters the structure and mechanical properties of cartilage, promotes chondrocyte damage, increases cartilage brittleness, and eventually results in progressive degeneration and secondary osteoarthritis-like changes (6). The spine is commonly affected early in the musculoskeletal course of the disease, followed by large joints such as the knees, hips, and shoulders. In advanced cases, patients may present with chronic back pain, spinal stiffness, progressive joint pain, restricted motion, and functional limitation.

Biochemical confirmation of alkaptonuria is based on elevated urinary homogentisic acid, and molecular confirmation can be obtained by identifying pathogenic variants in HGD. In the present case, urinary homogentisic acid quantification and HGD genetic testing were not performed, which is an important limitation. Nevertheless, the long-standing history of urine darkening after standing, characteristic spinal imaging changes, intraoperative black-brown pigmentation, and histopathological ochronotic pigment deposition strongly supported the clinicopathological diagnosis (7). Ochronotic arthropathy may mimic ordinary degenerative osteoarthritis in older adults. The diagnosis may be overlooked when clinicians focus only on local degenerative joint disease (8). However, careful history-taking and physical examination may reveal important diagnostic clues, including urine darkening after standing, pigmentation of the auricles or sclerae, spinal stiffness, and multi-joint involvement. Intraoperative black-brown pigmentation of cartilage, synovium, tendons, or joint capsule should also alert surgeons to the possibility of ochronosis (9). This patient had atypical features, including urine darkening, spinal involvement, multi-joint disease, and intraoperative pigmentation.

The pathological findings in ochronotic arthropathy should be distinguished from pigmented villonodular synovitis or tenosynovial giant cell tumor. Pigmented villonodular synovitis is a distinct proliferative synovial disorder characterized by synovial proliferation, hemosiderin deposition, mononuclear inflammatory infiltration, and multinucleated giant cells. In contrast, the pigmentation observed in ochronotic arthropathy results from ochronotic pigment deposition related to homogentisic acid metabolism rather than hemosiderin accumulation (10). In the present case, histopathological examination showed chronic synovitis with abundant brown-black ochronotic pigment deposition and focal lymphocytic infiltration, without definitive pathological features of pigmented villonodular synovitis or tenosynovial giant cell tumor. These findings were consistent with ochronotic arthropathy rather than true pigmented villonodular synovitis (11).

For end-stage ochronotic arthropathy with severe pain and functional limitation, total joint arthroplasty remains an effective treatment option. Although total knee and hip arthroplasty for ochronotic arthropathy has been described in previous case reports, several perioperative considerations are worth emphasizing. Surgeons should be aware that ochronotic pigmentation may involve not only articular cartilage and synovium, but also tendons, ligaments, the joint capsule, and other periarticular soft tissues. Pigmented cartilage and soft tissues may be degenerative, brittle, or fragile, and careful soft-tissue handling is therefore required during arthroplasty. Intraoperative black-brown discoloration should not be misinterpreted as infection, metallosis, or pigmented villonodular synovitis. In addition, because alkaptonuria is a systemic metabolic disorder, preoperative evaluation should not be limited to the affected joint, but should also consider spinal stiffness, urinary stone disease, cardiovascular valve involvement, and other systemic manifestations when clinically indicated (12).

In the present case, staged total knee arthroplasty and total hip arthroplasty resulted in marked pain relief and functional improvement. The Hospital for Special Surgery knee score improved from 40 preoperatively to 92 at five years after total knee arthroplasty, and the Harris hip score improved from 43 preoperatively to 94 at five years after total hip arthroplasty. At more than five years of follow-up, radiographs showed that the left knee and left hip prostheses remained well positioned, without evidence of loosening. The patient reported minimal pain, had a normal gait, and was independent in daily living. These findings suggest that staged arthroplasty may provide durable functional improvement in older patients with advanced ochronotic arthropathy.

Current management of alkaptonuria includes symptomatic treatment, pain control, physical therapy, monitoring for systemic involvement, and genetic counseling. Disease-modifying therapies such as nitisinone have been investigated as homogentisic acid-lowering treatments; however, established end-stage joint destruction may still require surgical intervention. Therefore, early recognition of alkaptonuria is valuable not only for explaining atypical multi-joint degeneration, but also for guiding systemic evaluation, patient education, long-term monitoring, and family counseling. In patients with advanced ochronotic arthropathy, arthroplasty remains an important option for pain relief and functional restoration.

This case report has several limitations. First, urinary homogentisic acid quantification and HGD genetic testing were not performed; therefore, the diagnosis was based on clinical history, urine discoloration after standing, characteristic imaging findings, intraoperative black-brown pigmentation, and histopathological evidence of ochronotic pigment deposition. This is an important limitation, and biochemical or genetic confirmation should be performed when available. Second, this is a single case report, and the findings cannot be generalized to all patients with ochronotic arthropathy. Third, although the patient had satisfactory function of the operated joints at the final follow-up, degenerative changes were also observed in the contralateral knee and hip, indicating the need for continued long-term monitoring. Nevertheless, the five-year follow-up suggests that staged total knee and hip arthroplasty may provide durable pain relief and functional improvement in advanced ochronotic arthropathy.

## Conclusion

Ochronotic arthropathy should be considered in older adults with atypical multi-joint degenerative changes, particularly when there is a history of urine darkening after standing, characteristic spinal involvement, or intraoperative black-brown pigmentation of cartilage and synovium. Histopathological confirmation of ochronotic pigment deposition is helpful, although biochemical and genetic testing should be performed when available. In advanced joint destruction, staged total knee and total hip arthroplasty can provide durable pain relief and functional improvement.

## Data Availability

The raw data supporting the conclusions of this article will be made available by the authors, without undue reservation.
